# Chinese Physiology

**Published:** 1847-04

**Authors:** 


13. *Chinese Physiology.*—"In medicine," says Mr. Williams, in his lecture on China, "the Chinese practice is better than their theory. The knowledge of some drugs enables them to effect occasional cures; but the Chinese have never dissected the human frame, and therefore know nothing of its anatomy. They assert that the food goes through the heart to the stomach—and further, that there are three avenues through the body!"

